# Cardiovascular Risk Profile and Lipid Management in the Population-Based Cohort Study LATINO: 20 Years of Real-World Data

**DOI:** 10.3390/jcm11226825

**Published:** 2022-11-18

**Authors:** Cristina Gavina, Daniel Seabra Carvalho, Marisa Pardal, Marta Afonso-Silva, Diana Grangeia, Ricardo Jorge Dinis-Oliveira, Francisco Araújo, Tiago Taveira-Gomes

**Affiliations:** 1 Cardiology Department, Pedro Hispano Hospital, Senhora da Hora, 4464-513 Matosinhos, Portugal; 2 Department of Medicine, Faculty of Medicine, University of Porto, 4200-319 Porto, Portugal; 3 Department of Community Medicine, Information and Decision in Health, Faculty of Medicine, University of Porto, 4050-313 Porto, Portugal; 4 Novartis Farma, Produtos Farmacêuticos S.A., 2740-255 Porto Salvo, Portugal; 5 MTG Research and Development Lab, 4200-604 Porto, Portugal; 6 TOXRUN–Toxicology Research Unit, Institute of Health Sciences, Advanced Polytechnic and University Cooperative (CESPU), CRL, 4585-116 Gandra, Portugal; 7 UCIBIO-REQUIMTE, Laboratory of Toxicology, Department of Biological Sciences, Faculty of Pharmacy, University of Porto, 4050-313 Porto, Portugal; 8 Department of Public Health and Forensic Sciences, and Medical Education, Faculty of Medicine, University of Porto, 4200-319 Porto, Portugal; 9 Hospital Lusíadas, 1500-458 Lisboa, Portugal; 10 Center for Health Technology and Services Research (CINTESIS), 4200-450 Porto, Portugal; 11 Faculty of Health Sciences, University Fernando Pessoa (FCS-UFP), 4249-004 Porto, Portugal

**Keywords:** CV risk categories, ASCVD, LDL-C management, dyslipidemia, real-world evidence

## Abstract

The rising prevalence of cardiovascular (CV) risk factors in Portugal has translated into more than 35,000 annual deaths due to CV diseases. We performed a multicenter observational cohort study encompassing clinical activities performed between 2000 and 2019 to characterize the CV risk profile and LDL-C management of patients in every CV risk category using electronic health records of a regional population in Portugal. We analyzed data from 14 health centers and 1 central hospital in the north of Portugal of patients between 40 and 80 years that had at least 1 family medicine appointment at these institutions. Living patients were characterized on 31 December 2019. CV risk assessment was computed according to the 2019 ESC/EAS Guidelines. Lipid-lowering therapy (LLT) and achievement of LDL-C targets were assessed. In total, the analysis included 78,459 patients. Patient proportions were 33%, 29%, 22%, and 17% for low, intermediate, high, and very high CV risk, respectively. Moderate-intensity statins were the most frequently used medication across all CV risk categories. High-intensity statins were used in 5% and 10% of high and very high CV risk patients, respectively. Ezetimibe was used in 6% and 10% of high and very high CV risk patients, respectively. LDL-C targets were achieved in 44%, 27%, 7%, and 3% of low, intermediate, high, and very high CV risk patients, respectively. For uncontrolled patients in the high and very high CV risk categories, a median LDL-C reduction of 44% and 53%, respectively, would be required to meet LDL-C targets. There are clear opportunities to optimize LDL-C management in routine clinical practice. The prescription of LLT according to CV risk represents an important missed treatment opportunity.

## 1. Introduction

The rising prevalence of cardiovascular (CV) risk factors in Portugal has translated into more than 35,000 annual deaths due to CV diseases, representing 29% of the total mortality in 2017 [1,2]. With stroke and ischemic heart disease as the leading causes of morbidity and death, reducing the incidence of atherosclerotic CV disease (ASCVD) is a major public health priority [3]. Recent studies confirmed the high burden and costs of atherosclerosis in Portugal, being responsible for 14% of all deaths and 12% of overall years of life lost (YLL) in 2016 while related direct and indirect costs were estimated at 1.9 billion euros per year (11% of Portuguese healthcare expenditure) [4,5]. Diseases and Injuries Collaborators. Global Burd The 2019 European Society of Cardiology (ESC)/European Atherosclerosis Society (EAS) Guidelines [6] recommend the assessment of cardiovascular risk using the Systematic Coronary Risk Evaluation (SCORE) system for adults >40 years of age, except for people with documented ASCVD, type 1 diabetes mellitus (T1D) or type 2 diabetes mellitus (T2D), chronic kidney disease (CKD), familial hypercholesterolemia (FH), or very high levels of individual risk (carotid or femoral plaques, coronary artery calcium score >100, or extreme Lp(a) elevation), which are automatically at high or very high CVD risk and should be managed accordingly.

Lowering of low-density lipoprotein cholesterol (LDL-C) is paramount to halt atherosclerotic plaque progression [7]. Thus, dyslipidemia has become a primary target for the prevention of atherosclerotic CV events [7,8]. A recent epidemiological study conducted in mainland Portugal estimated the prevalence of dyslipidemia by adapting the LDL-C reference levels from the NCEP-ATP III 2002 criteria and reported that 31.5% and 51.5% of the population aged 18–79 years had LDL-C >160 mg/dL and LDL-C >130 mg/dL, respectively [1,2]. Still, little is known regarding patient characteristics, lipid-lowering therapy (LLT), and LDL-C levels according to the CV risk categories used in current clinical practice.

This study aimed to describe the clinical characteristics, LLT, and control of LDL-C of patients according to the CVD risk categories defined in the 2019 ESC/EAS Guidelines, by leveraging the electronic health records produced in a regional population from northern Portugal over 20 years.

## 2. Materials and Methods

### 2.1. Study Design and Participant Selection

This was an observational cohort study using the electronic health records (EHRs) of patients followed at the Unidade Local de Saúde de Matosinhos (ULSM). ULSM is a large healthcare institution that includes 14 primary care centers assisted by 1 hospital that provides secondary and tertiary care services to the region of Matosinhos, reflecting the activity of more than 1000 doctors from different specialties. We considered a 20-year time window from 1 January 2000 to 31 December 2019 to scan EHRs for eligible patients. In order for a patient to be included in this study, the following criteria had to be met at the same point in time: (i) age between 40 and 80 years; (ii) at least one appointment with a ULSM primary care physician in the 3 years preceding the index date; and (iii) at least one record in the last year before the index date. The index date was defined as the calendar day of 31 December 2019. A total of 78,459 patients had enough EHR data to classify them in one category of CV risk and were thus characterized at the index date. The definition of the criteria was designed to be in line with the official government indicator used to determine whether a patient is routinely followed or not [9]. These inclusion criteria maximize the overlap of the study population with the resident population, which we believe to account for approximately 90% of the resident population of Matosinhos, according to the 2021 Portuguese Census [10]. Matosinhos is the eighth most inhabited municipality in the country and the fourth in the northern region [10].

This study was approved by the Ethical Committee and Data Protection Officer of ULSM (translated from Comissão de Ética para a Saúde da Unidade Local de Saúde de Matosinhos) under the approval codes No. 21/CE/JAS of 12 February 2021. All data processing and analysis were performed exclusively by analytic programs developed for this purpose and sent for execution at the ULSM data center. No data was extracted outside ULSM, and no direct access to the data took place. As an additional degree of security, processed data were de-identified by the ULSM Information Technology Department prior to the analytic code execution according to the Health Insurance Portability and Accountability Act (HIPAA) safe harbor standard [11]. 

### 2.2. Patient Characterization

All relevant conditions were identified using the ICPC-2, International Classification of Disease, 9th Revision (ICD-9) and International Classification of Disease, 10th Revision (ICD-10) codes. CV and diabetes medications were registered according to the Anatomical Therapeutic Chemical Classification System. Whenever possible, all patient conditions and criteria for risk assessment were reconstructed using the most granular available records of clinical measurements and laboratory results recorded at ULSM. Since DNA-based evidence was not available, FH was classified as definite (defined using ICD-10 code E78.01 and ICD-9 code 272.0) or possible (defined by ICD-10 code Z83.42 OR (TC > 290 mg/dL OR LDL-C >190 mg/dL AND (mother or father family history of TC >290 mg/dL OR family history of early heart disease in mother, ≤55 years-old, or father, ≤65 years-old (ICPC-2, ICD-9 or ICD-10 codes for myocardial infarction or unstable angina))) according to the Simon and Broome criteria [12,13]. In order to compute the family history of relevant diseases, we reconstructed familial relationships from primary care family information. We did not use carotid or coronary imaging data nor ankle brachial index, as this was not available. Patient demographic and clinical characteristics were described for the total cohort and for each CVD risk category. CVD risk assessment was computed according to the 2019 ESC/EAS Guidelines for the management of dyslipidemias [6]. Source data was harmonized according to the Observational Medical Outcomes Partnership (OMOP) Common Data Model (CDM) version 5.3 [14].

### 2.3. LDL-C Levels, Lipid-Lowering Therapy, and LDL-C Target Achievement

Patient LDL-C level was defined as the LDL-C value reported in the latest available laboratory test relative to the index date. When LDL-C values were not available, we computed them using the Friedewald formula [15]. The proportion of patients under any LLT and specifically treated with statins, ezetimibe, fibrates, combination therapy of ezetimibe with statins, and others was assessed for each CVD risk category. The intensity of statin therapy (low, moderate, or high) was also assessed. Since PCSK-9 inhibitors were not available for use in ULSM within the study period they were not considered for the analysis.

To ascertain LDL-C target achievement, we considered ESC/EAS 2019 risk-based recommended targets: ≤116 mg/dL for low risk; <100 mg/dL for intermediate risk; <70 mg/dL for high risk; and <55 mg/dL for very high risk [6]. The same analysis was performed considering the ESC/EAS 2016 targets [16]. The proportion of patients achieving the ESC/EAS 2019 and 2016 LDL-C targets was calculated for each CVD risk category and according to LLT.

### 2.4. LLT Exposure

LLT exposure was computed from medication records filed by ULSM physicians in both primary and secondary care. Individual drugs were mapped to LLT categories using the Anatomical Therapeutic Chemical Classification System (ATC). The potency group, considering both the drug and the dosage, was attributed according to the classification of the American College of Cardiology and the American Heart Association [17]. We considered that a patient was under treatment for a given LLT category if the patient had been prescribed one LLT drug within 365 days from the index date.

### 2.5. Statistical Analysis

All analyses were descriptive with no comparison within patient groups. Continuous variables were reported as the mean and standard deviation (SD) or median and interquartile range (IQR). Categorical variables were presented as frequency counts and percentages. Missing data was considered a separate level and described through frequency counts and percentages. Apache Spark Framework version 2.4.5 and R version 4.0.3 were used to perform the statistical analysis.

## 3. Results

### 3.1. Patient Characteristics and CVD Risk Profile

We identified 81,727 eligible patients, from which 4% were excluded due to either no record of LDL-C measurement or an absence of enough information to stratify into any CVD risk category. A total of 78,459 patients were included in the analysis. The mean (SD) age of the overall cohort was 59 (11) years and 42% of the patients were male. The most common comorbidities were hypercholesterolemia (50%) and hypertension (50%), followed by obesity (25%) and T2D (17%). Approximately 1% of the patients had FH (definite or possible). A detailed characterization of the cohort is shown in Table 1.

According to the 2019 ESC/EAS Guidelines definitions for CVD risk stratification, 33%, 29%, 22%, and 17% of the patients had low, intermediate, high, and very high CVD risk, respectively. 

Within the 39% of the patients with high or very high risk, 57% were male and the mean (SD) age was 59 (19) years. Hypertension was the most prevalent comorbidity in both high (66%) and very-high-risk (79%) patients, followed by hypercholesterolemia (51% and 46%, respectively). Considering very-high-risk patients, 63% had T2D and 38% had ASCVD. Within ASCVD patients, the most common type was ischemic stroke (63%), followed by myocardial infarction (33%), peripheral artery disease (PAD) (11%), and unstable angina (6%). 

Considering ASCVD patients who were treated with any LLT (n = 4428), 14% were treated with a high-intensity statin and 11% with ezetimibe. A significant proportion of patients in the high and very high CV risk categories were not receiving any LLT (27% and 15%, respectively). At the time of this analysis, no patients were under proprotein convertase subtilisin/kexin type 9 inhibitors (PCSK9i). Moderate-intensity statins were the most frequently used LLT for all risk categories, followed by low-intensity statins. The proportion of patients using high-intensity statins was smaller, ranging from 4% to 12% for the low and very high CV risk categories respectively. More detailed information on LLT use by CVD risk category is shown in Table 2.

### 3.2. LDL-C Levels and LDL-C Targets Achieved by CVD Risk

The study population had a median (IQR) LDL-C of 117 (52) mg/dL. Median LDL-C levels differed according to the CVD risk category, being lower in the low-risk group (115 (46) mg/dL) and higher in the high-risk group (122 (70) mg/dL). Considering ASCVD patients, the median (IQR) LDL-C was 105 (55) mg/dL. Table 3 summarizes the lipid values in the overall population and by CVD risk category.

**Figure 1:**
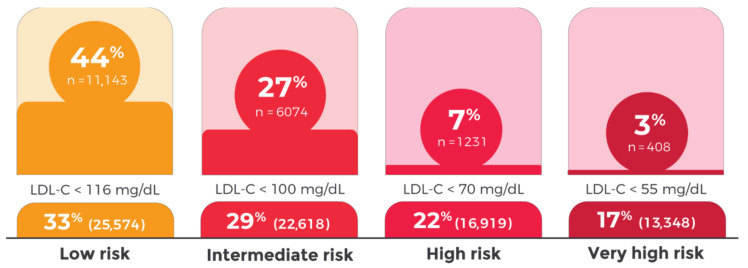
Low-density lipoprotein cholesterol (LDL-C) control by cardiovascular risk category, 2019 ESC/EAS Guidelines.

Despite the described LLT use, only 24% of the patients achieved the LDL-C targets set by the 2019 ESC/EAS Guidelines for the management of dyslipidemias. In patients with very high CVD risk, only 3% achieved LDL-C levels <55 mg/dL (5% in patients with ASCVD). With respect to patients with high, intermediate, and low CVD risk, 7%, 27%, and 44% achieved their CVD level LDL-C targets, respectively (Figure 1).

Considering the uncontrolled patients in each CVD risk category, the median relative LDL-C reduction required to meet the LDL-C target according to the 2019 ESC Guidelines for the management of dyslipidemias was 15%, 25%, 44%, and 53% for the low, intermediate, high, and very high CV risk categories, respectively. Considering the 2016 ESC/EAS Guidelines, 34% of the patients achieved their LDL-C targets (10%, 28%, 42%, and 44% of the patients in the very high, high, intermediate, and low CVD risk categories, respectively).

## 4. Discussion

This cross-sectional study showed that more than one in every three individuals followed at ULSM were determined to be in either the high or very high CV risk categories, yet they had very poor control of LDL-C. Only 7% of the high-risk patients and 3% of the very-high-risk patients reached the ESC/EAS 2019 LDL-C targets. Moderate-intensity statins were the most frequently used LLT, irrespective of the CV risk category, and a substantial proportion of patients had no LLT despite meeting the criteria to start medication.

LDL-C is a key determinant of CVD risk and multiple studies have documented a linear relationship between LDL-C levels and CV events [8], underscoring LDL-C as the primary treatment target in clinical recommendations [6,8]. Moreover, a reduction in LDL-C is clearly linked to a substantial decrease in the risk of major adverse cardiovascular events and CV death [18,19].

We characterized more than 95% of the eligible patients at ULSM. Considering the high usage rate of ULSM by the resident population, the low population migration rates, and the large data collection period spanning more than a decade, we believe that these findings can be generalized to the population served in this region, and to populations of a similar composition. This is further substantiated by the correspondence between the sex distribution for the age groups under analysis for the Matosinhos population and the overall Portuguese population [10].

Patients with high and very high risk accounted for 39% of the studied population. Particularly regarding those at higher risk, a clear disparity between real-world routine clinical practice and the ESC/EAS 2019 guidelines was observed regarding LDL-C control and LLT prescription. A substantial proportion of high- and very-high-risk patients have never received any LLT. This proportion is similar to what was found in a recent study [20] that assessed temporal trends in the use of LLT in very-high-risk patients followed in the Cardiology Department of the Central University Hospital and concluded that about one-third of the study population was not receiving any LLT [21,22,23]. These findings underscore the need to support clinicians to systematically identify factors other than the SCORE that allow for direct stratification in high or very high CVD risk. The preferential use of moderate-intensity statins LLT irrespective of the CV risk category represents an important missed treatment opportunity and a likely avenue to significantly improve LDL-C control in high- and very-high-risk patients.

LDL-C target achievement was suboptimal as only 24% of the patients attained the corresponding 2019 ESC/EAS guideline target. The LDL-C target was more frequently achieved in patients at lower risk, and only 3% of the very-high-risk patients had an LDL-C value that was within the recommended target. These results are in line with previously published studies: (i) the DA VINCI study showed that only 33% of the patients followed in selected primary and secondary care European centers reached the LDL-C targets recommended by the 2019 ESC/EAS guidelines [24]; (ii) the EUROASPIRE survey reported that 29% of very-high-risk patients had reached the 2016 ESC/EAS LDL-C targets [25]; and (iii) Araújo et al. [20] reported a LDL-C control of 24% in a specialized tertiary center. Ours is a real-world study that is representative of the overall population followed in both primary and secondary care; thus, the degree of LDL-C control and adequate LLT use were expected to be lower. Based on these findings, we believe that studies conducted in this fashion will yield results that are closer to ours.

Several reasons may contribute to explain our findings: (i) Firstly, there was a high proportion of patients who were not receiving any LLT. Since those included in this study had at least one appointment with a ULSM family medicine physician in the three years preceding the index date, it was expected that at least in the high and very high CV risk categories, LLT would always be prescribed. This observation suggests that CV risk stratification with criteria other than SCORE and history of MACE may be difficult to perform; (ii) secondly, failure to optimize the use of high-intensity statins represented a major missed treatment opportunity, which likely explains a substantial proportion of the marginal degree of LDL-C control in high- and very-high-risk patients; and (iii) finally, residual use of ezetimibe is also expected to contribute substantially to the marginal degree of LDL-C control in high- and very-high-risk patients.

Despite this evidence, it is important to acknowledge that the 2019 ESC/EAS LDL-C targets for patients with high and very high risk are difficult to achieve with high-intensity statin monotherapy, which underscores the need to resort to combination therapies, especially with ezetimibe [6,8]. Our results showed a substantially higher proportion of fibrate use when compared to ezetimibe, which may be explained by the lack of awareness of the most recent recommendations in this area, and the growing evidence that fibrates may play a potential beneficial role in “atherogenic dyslipidemia”, which is particularly present in diabetic patients [26,27]. Previous CV outcome trials demonstrated that the risk reduction with fibrates tends to be proportional to the degree of non-HDL-C lowering [18]; moreover, the CV benefits of this therapy are ambiguous [28] and thus, they currently hold a class IIb recommendation in the 2019 ESC/EAS Guidelines [6]. There are ongoing studies to clarify the CV benefits of fibrates [29].

One of the strengths of our study resides in the fact that the EHR data used integrates primary, secondary, and tertiary health care units; thus, our cohort more closely represents the general CV risk population than cohorts from only one of those settings separately. Another strength is that we scanned all electronic data from the past 20 years, giving this analysis a very robust and detailed patient and family history. Regarding limitations, it must be mentioned that generalization of these results to dissimilar populations is limited as these data are relative to a specific population in the north of Portugal. However, the high degree of patient inclusion and the high rate of patient reconstruction of a stable regional population provides a detailed and unconstrained real-world perspective of patient populations under primary and secondary care followed in similar settings. Another limitation is that LLT medication may have been marginally overestimated as it was not possible to confirm whether prescriptions were filled.

In light of these results, one must consider whether: (i) the lack of awareness of recommendations is a factor contributing to suboptimal lipid management; (ii) treatment inertia may play a substantial role in reviewing LLT; (iii) patient beliefs and misconceptions regarding LLT may hinder therapy intensification; and (iv) patient CVD risk category stratification may be hard to perform in routine clinical practice that is expected to be performed in an incomplete fashion. The recently released 2021 ESC Guidelines on cardiovascular disease prevention in clinical practice position CVD risk estimation as the cornerstone for a tailored intervention at the individual level, with a stepwise approach to risk factor treatment and treatment intensification. This includes apparently healthy people, patients with established ASCVD, patients with T2D, and patients with specific risk factors such as CKD and FH [30]. Thus, it becomes paramount to study the impact of the enumerated considerations to increase the real-world applicability of the recommendations.

## 5. Conclusions

Our study identified major missed treatment opportunities to optimize LDL-C management for every CVD risk category, with particular emphasis on high- and very-high-risk patients. It also highlighted the need to use high-intensity statins and combination therapy, putting the most recent recommendations into clinical practice. In this regard, investment in adequate control of LDL-C seems to be the most promising solution to decrease the high burden of ASCVD in Portugal.

## Figures and Tables

**Table 1:** Sociodemographic and clinical characteristics by cardiovascular risk category on 31 December 2019.

	Overall N = 78,459	Low n = 25,574	Intermediate n = 22,618	High n = 16,919	Very High n = 13,348
Female, n (%)	45,328 (58)	18,951 (74)	12,833 (57)	7868 (47)	5676 (43)
Age (years), mean (SD)	59 (11)	47 (5)	61 (7)	67 (10)	67 (10)
Systolic blood pressure (mmHg), median (IQR)	132 (18)	125 (18)	133 (16)	136 (17)	137 (19)
Diastolic blood pressure (mmHg), median (IQR)	80 (12)	80 (12)	81 (12)	80 (12)	80 (13)
Body mass index (Kg/m^2^), median (IQR)	27 (6)	26 (6)	27 (6)	27 (6)	28 (6)
Waist circumference (cm), median (IQR)	97 (15)	92 (17)	96 (15)	99 (14)	101 (15)
Smoking history, n (%)					
Never	58,388 (74)	18,870 (74)	16,167 (72)	13,480 (80)	9871 (74)
Current	14,379 (18)	4797 (19)	5055 (22)	2050 (12)	2477 (19)
Former	3142 (4)	654 (3)	907 (4)	883 (5)	698 (5)
Unknown	2550 (3)	1253 (5)	489 (2)	506 (3)	302 (2)
Comorbidities, n (%)					
Hypertension	39,305 (50)	5859 (23)	11,775 (52)	11,140 (66)	10,531 (79)
Hypercholesterolemia	39,593 (50)	12,552 (49)	12,290 (54)	8574 (51)	6177 (46)
Obesity	19,513 (25)	5045 (20)	5335 (24)	4658 (28)	4475 (34)
Type 2 diabetes mellitus	13,464 (17)	120 (0.5)	229 (1)	4653 (28)	8462 (63)
Structural heart disease	10,462 (13)	1098 (4)	1859 (8)	2265 (13)	5240 (39)
Microvascular disease	4019 (5)	697 (3)	854 (4)	725 (4)	1743 (13)
Stable angina	1957 (2)	15 (0.1)	94 (0.4)	173 (1)	1675 (13)
Atrial fibrillation	1805 (2)	49 (0.2)	304 (1)	420 (2)	1032 (8)
Chronic kidney disease	4313 (6)	0 (0)	0 (0)	1669 (10)	2644 (20)
Familial hypercholesterolemia	767 (1)	0 (0)	0 (0)	46 (0.3)	721 (5)
Definite	746 (1)	0 (0)	0 (0)	45 (0.3)	701 (5)
Possible	23 (0)	0 (0)	0 (0)	2 (0)	21 (0.2)
Cardiovascular disease	43,222 (55)	6100 (24)	12,298 (54)	12,113 (72)	12,711 (95)
Atherosclerotic cardiovascular disease	5088 (6)	0 (0)	0 (0)	0 (0)	5088 (38)
Ischemic stroke	3207 (4)	0 (0)	0 (0)	0 (0)	3207 (24)
Peripheral artery disease	569 (1)	0 (0)	0 (0)	0 (0)	569 (4)
Myocardial infarction	1665 (2)	0 (0)	0 (0)	0 (0)	1665 (12)
Unstable angina	309 (0.4)	0 (0)	0 (0)	0 (0)	309 (2)

IQR: interquartile range; SD: standard deviation.

**Table 2:** Lipid-lowering therapy use, overall and by cardiovascular risk category on 31 December 2019.

	Overall N = 78,299	Low n = 25,718	Intermediate n = 23,357	High n = 12,857	Very High n = 16,367
	n (%)	n (%)	n (%)	n (%)	n (%)
Any LLT	40,281 (52)	4902 (19)	13,065 (56)	9152 (71)	13,162 (80)
Statin	38,990 (50)	4566 (18)	12,662 (54)	8851 (69)	12,911 (79)
Low-intensity statin	3163 (8)	416 (9)	1094 (9)	804 (9)	849 (7)
Moderate-intensity statin	32,965 (85)	3973 (87)	10,933 (86)	7594 (86)	10,465 (81)
High-intensity statin	2862 (7)	177 (4)	635 (5)	453 (5)	1597 (12)
Ezetimibe + Statin	3202 (4)	226 (1)	849 (4)	651 (5)	1476 (9)
Ezetimibe + Low-intensity statin	234 (7)	24 (11)	79 (9)	59 (9)	72 (5)
Ezetimibe + Moderate-intensity statin	2482 (78)	193 (85)	678 (80)	515 (79)	1096 (74)
Ezetimibe + High-intensity statin	486 (15)	9 (4)	92 (11)	77 (12)	308 (21)
Ezetimibe mono	3335 (4)	244 (1)	888 (4)	684 (5)	1519 (9)
Fibrates	7903 (10)	917 (4)	210 (9)	1824 (14)	3059 (19)
Fibrates + Statin	2906 (3)	323 (1)	688 (3)	650 (4)	1215 (7)
PCSK9 inhibitors	0 (0)	0 (0)	0 (0)	0 (0)	0 (0)
Other	170 (0)	18 (0)	51 (0)	31(0)	70 (0)

PCSK9: proprotein convertase subtilisin/kexin type 9.

**Table 3:** Lipid panel for patients in every cardiovascular risk category on 31 December 2019.

	Overall N = 78,299	Low n = 25,718	Intermediate n = 23,357	High n = 12,857	Very High n = 16,367
	median (IQR)	median (IQR)	median (IQR)	median (IQR)	median (IQR)
LDL-C, mg/dL	118 (52)	114 (47)	116 (48)	126 (80)	120 (60)
HDL-C, mg/dL	48 (16)	51 (17)	49 (15)	47 (16)	45 (16)
Non-HDL-C, mg/dL	144 (48)	142 (41)	144 (47)	148 (54)	146 (58)
TC, mg/dL	195 (50)	194 (43)	193 (51)	198 (56)	195 (60)
TG, mg/dL	102 (66)	86 (54)	100 (58)	114 (77)	120 (80)

HDL-C: high-density lipoprotein cholesterol; IQR: interquartile range; LDL-C: low-density lipoprotein cholesterol; Non-HDL-C: non- high-density lipoprotein cholesterol; TC: total cholesterol; TG: triglycerides.

## Data Availability

All aggregate statistical results are incorporated into this article. Patient level data used in this study is not publicly available.
